# Human-Derived Cellular Models in Psychiatry: A Focus on the Olfactory Neuroepithelium

**DOI:** 10.3390/brainsci16050523

**Published:** 2026-05-14

**Authors:** Tommaso Toffanin, Mario Angelo Pagano, Carlo Idotta, Luigi Grassi, Anna Maria Brunati

**Affiliations:** 1 Department of Neuroscience and Rehabilitation, Institute of Psychiatry, University of Ferrara, Via Fossato di Mortara, 64/A, 44121 Ferrara, Italy; pgnmng@unife.it (M.A.P.); luigi.grassi@unife.it (L.G.); 2 Metis ETS, 35018 San Martino di Lupari, Italy; carloidottapsi@gmail.com; 3 Department of Molecular Medicine, University of Padova, 35121 Padova, Italy; annamaria.brunati@unipd.it

**Keywords:** psychiatric disorders, post-mortem brain studies, human embryonic stem cells, induced pluripotent stem cells, olfactory neuroepithelium

## Abstract

**Highlights:**

**What are the main findings?**

• Research on the biological basis of mental disorders has progressively evolved from post-mortem and animal studies to human-derived cellular models, enabling increasingly mechanistic and patient-relevant insights.
• Converging evidence across models reveals shared neurodevelopmental, synaptic, and molecular alterations underlying major psychiatric disorders beyond traditional diagnostic boundaries.

**What are the implications of the main findings?**

• Integrating complementary models is essential to capture the multilevel complexity of psychiatric diseases, from genes to circuits to clinical phenotypes.
• While all models contribute to deciphering the biology of mental disorders, the olfactory neuroepithelium—by virtue of its accessibility and scalability—appears particularly promising for enabling large-cohort and longitudinal studies, thereby accelerating biomarker discovery and the development of precision psychiatry approaches.

**Abstract:**

Severe mental disorders, including schizophrenia (SCZ), bipolar disorder (BD), and major depressive disorder (MDD), are leading causes of global disability, yet current treatments remain largely symptomatic and fail to alter disease trajectories. Converging evidence from genetics, longitudinal studies, and systems neuroscience supports a dimensional and transdiagnostic architecture of psychopathology, involving shared polygenic risk and overlapping neurodevelopmental and circuit-level alterations. Traditional approaches—such as post-mortem brain analysis, neuroimaging, and animal models—have delineated core molecular perturbations (e.g., dopaminergic, glutamatergic, and GABAergic dysfunction), as well as informed translational frameworks for mechanistic investigation, but remain constrained by restricted access to dynamic processes and incomplete recapitulation of human-specific biology. The advent of human-derived cellular models, particularly human embryonic stem cells (hESCs) and induced pluripotent stem cells (iPSCs), has partially addressed these limitations, enabling the study of patient-specific neurodevelopment and synaptic function in vitro. Within this evolving landscape, the olfactory neuroepithelium (ONE) has emerged as an accessible source of neural progenitors, obtainable through minimally invasive procedures, providing a window into living human neurobiology. ONE-derived cells retain donor-specific genetic and epigenetic signatures while recapitulating disease-relevant phenotypes across major psychiatric disorders, including altered neurodevelopmental dynamics, synaptic gene expression, and inflammatory profiles. Here, we present a narrative review of the principal cellular and tissue models used in biological psychiatry, examining their respective strengths, limitations, and translational relevance across experimental contexts. By situating these approaches within a unified framework, we aim to clarify their complementarity, identify current gaps, and outline future directions, highlighting the emerging potential of ONE-based models to bridge genetic risk, cellular dysfunction, and clinical phenotype, thereby advancing precision psychiatry.

## 1. Introduction

Severe mental disorders—including schizophrenia (SCZ), bipolar disorder (BD), and major depressive disorder (MDD)—represent a major global public health challenge and are among the largest contributors to the global burden of disease, particularly in terms of years lived with disability worldwide [1,2,3]. Together, these disorders account for a disproportionate share of health-care expenditure, with lifetime costs per patient exceeding those of cancer and cardiovascular disease in many countries [2,4,5]. Importantly, severe mental disorders profoundly disrupt social functioning, employment, and quality of life, generating substantial indirect costs related to productivity loss, caregiving demands, and reliance on social welfare systems [5,6]. Despite the high prevalence and societal impact of mental disorders, the currently available treatments remain largely symptomatic and fail to meaningfully impact clinical outcomes and disease course [7,8], with persistent cognitive impairment, functional decline, disease progression, or relapse [7,9]. These limitations highlight the urgent need to better understand the mechanisms underlying the pathogenesis of mental disorders and identify novel biological targets for treatment [7,10].

Accumulating evidence indicates that mental disorders arise from complex interactions among genetic vulnerability, aberrant neurodevelopmental trajectories, and environmental exposures [8,10,11,12,13]. Large-scale genome-wide association studies (GWAS) have identified hundreds of shared risk loci across SCZ, BD, and MDD that converge on synaptic, immune, and neurodevelopmental pathways [14,15,16,17,18]. This shared genetic architecture extends to neurodevelopmental disorders such as autism spectrum disorder (ASD) and attention-deficit/hyperactivity disorder (ADHD), suggesting a highly interconnected biological landscape that challenges traditional diagnostic boundaries [17,18,19].

Consistent with this convergence, polygenic risk analyses and longitudinal studies support a continuum of biological liability, indexed by the so-called “p-factor,” a latent dimension capturing shared, transdiagnostic genetic risk across internalising, externalising, and psychotic domains [20,21].

Understanding severe mental disorders requires experimental frameworks that integrate genetic risk, neurodevelopment, cellular pathology, brain circuitry, and clinical phenotypes [6,8,18,22,23,24]. Established approaches—including post-mortem human brain studies, in vivo neuroimaging, and animal models—have been essential for identifying alterations in cortical architecture, synaptic and glial organisation, large-scale network dysfunction, and causal mechanisms of risk [25,26,27,28,29,30,31,32]. Recent large-scale meta-analyses and advanced neuroimaging frameworks have further refined our understanding of these alterations, mapping robust patterns of cortical thinning and subcortical volume loss across thousands of patients [7,33,34,35,36].

However, these approaches are limited by their reliance on static endpoints, indirect measures of cellular pathology, or incomplete translation to human disease biology, therefore failing to fully capture the complexity, developmental origins, and patient-specific nature of psychiatric disorders. In response to these limitations, human-derived cellular platforms—including induced pluripotent stem cell (iPSC)-based neural models and olfactory neuroepithelium (ONE)-derived cells—have emerged as valuable tools for investigating patient-specific investigation of neurodevelopmental, synaptic, and molecular phenotypes in SCZ, BD, and related conditions [37,38,39,40,41,42,43,44,45].

This narrative review synthesises the principal biological models used in psychiatry, outlining their strengths, limitations, and translational scope. Within this framework, these models are best regarded as complementary and synergistic rather than hierarchically ordered. In this context, we position the olfactory neuroepithelium (ONE) as a human-derived cellular platform that not only complements established approaches but also holds distinct translational potential, providing access to patient-specific neurobiology and enabling the investigation of disease-relevant mechanisms. Integrating these models is therefore essential for achieving a more precise, biologically grounded understanding of the cellular and molecular mechanisms underlying mental disorders and for bridging genetic risk, cellular dysfunction, and clinical phenotype.

This review is intended for both basic researchers and clinician-scientists, with the hope of promoting knowledge exchange and fostering integration between experimental and clinical perspectives.

## 2. Review Methodology

This narrative review was conducted using an AI-assisted literature search strategy complemented by manual curation and full-text appraisal, without adherence to formal systematic review protocols. An initial screening of the scientific literature was performed using Consensus 2.0 (consensus.app), an artificial intelligence-based platform designed to retrieve and synthesise peer-reviewed scientific evidence. Searches were conducted using a combination of free-text terms, including “psychiatric disorders” OR “mental disorders”, “neurodevelopmental disorders”, “schizophrenia”, “major depressive disorder”, “bipolar disorder”, in combination with “cellular models”, “olfactory neuroepithelium” OR “olfactory mucosa”, “induced pluripotent stem cells” OR “iPSC”, “human embryonic stem cells” OR “hESC”, “MUSE cells”, and terms related to post-mortem tissue, animal models, and translational and precision psychiatry. References identified through Consensus were subsequently expanded and cross-validated using Scopus for broader coverage of related studies and citation networks. Collectively, this strategy yielded approximately 1200 potentially relevant references.

Retrieved articles were progressively screened and evaluated according to their relevance to the objectives of the review. Titles and abstracts were manually assessed by the authors to exclude records that were clearly off-topic, duplicated across databases, or unavailable in English. This process yielded 325 references, for which full-text articles were subsequently retrieved via PubMed/MEDLINE, with concurrent verification of bibliographic information and DOI indexing.

The 325 full-text articles then underwent further evaluation according to their conceptual and methodological contribution to the review objectives. Studies of primary importance underwent detailed full-text evaluation, whereas more peripheral articles were evaluated selectively according to their relevance to specific thematic sections. Priority was given to studies directly addressing cellular or tissue models in biological psychiatry, with particular reference to olfactory neuroepithelium, iPSC-derived systems, post-mortem tissue, and experimental animal models. Studies were excluded if they addressed psychiatric conditions only marginally, lacked substantial empirical or conceptual relevance to the aims of the review, were published as preprints or in non-peer-reviewed sources, or had been superseded by more comprehensive subsequent studies cited within the review. Application of these criteria resulted in the inclusion of 165 articles.

Particular emphasis was placed on studies published between 2010 and 2026, reflecting major advances in iPSC and olfactory neuroepithelium (ONE) technologies, although earlier foundational studies were included where directly relevant.

Given the narrative nature of this review, no formal quality assessment or risk-of-bias tools were applied. The potential for selection bias inherent to narrative synthesis is acknowledged. This review aims to provide an integrative and critical synthesis of current evidence rather than a quantitative meta-analysis.

## 3. Current Methodological Landscapes in Biological Psychiatry: From Post-Mortem Brains to Cell-Based Models

Post-mortem brain analysis remains the gold standard for identifying tissue, cellular, and molecular pathology in major psychiatric disorders, consistently demonstrating reduced cortical thickness and synaptic density in SCZ and BD [25], with reductions in dendritic spine density correlating with in vivo MRI evidence of grey matter volume loss [46]. Although ex vivo brain slices enable direct investigation of local circuit physiology, findings may be influenced by well-established confounding factors, including agonal state, post-mortem interval, medication exposure, and diagnostic variability [47,48,49,50]. Converging neuroimaging studies further reveal shared structural and functional abnormalities across SCZ, BD, and MDD, particularly within prefrontal and fronto-limbic networks, where cortical thinning follows a gradient of severity from MDD to BD to SCZ, supporting a dimensional neuroanatomical spectrum [30,31,51,52]. Post-mortem and cellular studies consistently report overlapping disturbances in cortical organisation, synaptic density, inhibitory interneuron function—especially parvalbumin-positive cells—dendritic spine morphology, synaptic plasticity, and mitochondrial metabolism across psychotic and mood disorders, with greater alterations associated with psychosis and cognitive impairment [25,29,53,54,55,56,57]. Molecular abnormalities identified in post-mortem human brain tissue, together with converging evidence from pharmacological interventions in patients, have underpinned landmark hypotheses regarding the aetiology of psychiatric disorders—most notably the dopaminergic hypothesis of SCZ and the monoaminergic hypothesis of depression [58,59,60]. Early post-mortem biochemical studies demonstrating increased dopamine D2 receptor density in the striatum of individuals with SCZ [58] provided a critical empirical foundation for these models and, more broadly, for the development of translational research strategies. Building on these findings, subsequent work in non-human primates, whose prefrontal cortical organisation closely resembles that of humans, has been instrumental in delineating the distinct contributions of dopamine D1 and D2 receptors to executive function and cognitive control, thereby informing the development and testing of antipsychotic compounds [61,62].

Post-mortem investigations have also driven the formulation of the glutamatergic and GABAergic hypotheses of SCZ. Evidence of reduced N-methyl-D-aspartate (NMDA) receptor expression in the hippocampus [25] led directly to the development of experimental models of NMDA receptor hypofunction, including genetic knockout mice that recapitulate key behavioural and neurochemical features of the disease [63]. Similarly, consistent findings of impaired GABAergic interneuron function—particularly decreased expression of parvalbumin (PV) and glutamic acid decarboxylase, i.e., the enzyme converting glutamic acid into gamma-amino butyric acid (GABA), in the prefrontal cortex [64]—have guided the generation of PV cell-specific models that reproduce deficits in gamma oscillations and working memory, thereby implicating inhibitory circuit dysfunction in the cognitive symptoms of SCZ [54].

These converging lines of evidence have collectively established a robust translational framework for in vivo modelling of psychiatric disorders. A range of complementary approaches—including genetic manipulation (e.g., DISC1 or NRXN1 mutations) [65,66], pharmacological challenges (e.g., ketamine-induced psychosis) [67], and environmental paradigms (e.g., maternal immune activation) [68]—have been implemented primarily in rodent systems, enabling the dissection of molecular and circuit-level mechanisms.

While these models offer the capacity to perform longitudinal studies, establish brain-behaviour correlations, and test novel therapeutics, they are constrained by an inherent evolutionary gap that hinders the exploration of complex psychiatric symptoms such as hallucinations, delusions, and guilt [27].

Non-human primate models, owing to their closer neuroanatomical resemblance to humans, partially bridge this gap but are limited by ethical concerns and substantial financial costs [61,62]. Building on the limitations of post-mortem and animal models, the field has progressively shifted toward human-derived cellular models. Among these, pluripotent stem cell technologies—initially human embryonic stem cells (hESCs) and later induced pluripotent stem cells (iPSCs)—have provided experimentally accessible platforms for generating human neural progenitors, neurons, and glial cells in vitro, enabling the investigation of early neurodevelopmental processes, patient-specific cellular phenotypes, and molecular pathways associated with psychiatric disorders, offering opportunities for disease modelling and drug screening, as discussed below.

## 4. Exploiting Human Cell Pluripotency: From Embryonic Stem Cells to Patient-Derived Models

### 4.1. Human Embryonic Stem Cells (hESCs)

Human embryonic stem cells (hESCs) were first derived from human blastocysts [69], following nearly two decades of extensive investigation in animal models, ranging from rodents to non-human primates [70,71,72]. hESCs possess pluripotency and replicative immortality [69,73], maintaining an undifferentiated state confirmed by characteristic surface markers (SSEA-3, SSEA-4, TRA-1-60, TRA-1-81, and alkaline phosphatase) and by teratoma formation assays demonstrating derivatives of all three germ layers in immunodeficient mice [69,74]. The capacity of hESCs to generate neural epithelium and diverse ectodermal derivatives provides a human platform for modelling early brain development [69,74]. The implementation of cell culture protocols directing the formation of neural precursors in vitro has enabled the generation of specified neural progenitors, as well as differentiated neurons and glial cells of distinct subtypes [75,76,77,78], supported by feeder-independent media, incorporating specific growth factors including bFGF and TGFβ [79,80].

Because many psychiatric disorders involve subtle neurodevelopmental trajectories that cannot be faithfully reproduced in rodents, hESC-derived neural lineages offer a biologically relevant system for dissecting differentiation programmes, synaptic maturation, and early pathogenic mechanisms [81]. Their scalability also supports high-throughput pharmacological screening to evaluate neurotoxicity and identify compounds affecting human neural differentiation [73,74].

Bioprocessing innovations such as automated tissue culture systems, embryoid body formation, and microcarrier-based expansion have enabled the transition from manual methods to scalable, high-throughput production [79,80]. Nonetheless, several limitations remain. The derivation of hESCs from the inner cell mass of human blastocysts raises ethical concerns related to embryo destruction and has prompted ongoing policy and funding debates [82,83]. However, reproducible large-scale differentiation into specific neural subtypes remains technically challenging [84]. Pluripotent stem cells carry intrinsic tumorigenic potential and may form teratomas if undifferentiated cells persist in transplantation products, necessitating stringent purification and safety controls [85]. Finally, hESC-derived cells are typically allogeneic and may trigger immune rejection after transplantation, requiring immunosuppression or alternative strategies to achieve immune compatibility [86].

### 4.2. Induced Pluripotent Stem Cells

The advent of induced pluripotent stem cell (iPSC) technology has overcome several ethical and practical limitations associated with hESCs and has provided an unprecedented platform for neuropsychiatric research. iPSCs were first generated by Shinya Yamanaka and colleagues through the ectopic expression of four transcription factors (OCT4, SOX2, KLF4, and c-MYC) in mouse differentiated somatic cells [87], which were subsequently adapted to adult human dermal fibroblasts [88]. These cells can be differentiated into multiple neuronal and glial subtypes—including dopaminergic, glutamatergic, and GABAergic neurons—as well as astrocytes and oligodendrocytes, providing experimentally accessible models for human brain development and disease [89,90,91]. In neuropsychiatric research, iPSC-based models have provided critical cellular and molecular insights. For instance, iPSC-derived neurons from patients with SCZ exhibit synaptic deficits, reduced dendritic arborisation, and altered calcium signalling, reflecting neurodevelopmental disruptions observed in vivo [37,92]. iPSCs from patients with BD differentiated into cortical neurons show hyperexcitability and differential responses to lithium, linking cellular electrophysiology to patient-specific pharmacological outcomes [38]. iPSC-derived neurons from individuals with MDD display altered serotonergic signalling and impaired network synchrony [93]. Organoid models of neurodevelopmental disorders such as ASD reveal abnormal neuronal proliferation, cortical layering defects, and dysregulated expression of synapse-associated genes, enabling mechanistic studies not possible in two-dimensional cultures [91,94]. Combined with genome editing, iPSC technology facilitates high-throughput chemical screening and the identification of transcriptional drug responses that reverse disease-associated signatures [95,96,97].

Despite these advances, several limitations persist. iPSCs retain epigenetic memory from their somatic cell of origin, which can bias differentiation efficiency and gene expression patterns [98,99]. The generation of iPSCs remains costly and time-consuming, requiring specialised reagents and culture conditions, as well as extensive quality control [100,101,102,103,104,105]. iPSC-derived neurons often exhibit a foetal-like developmental state and may not fully capture adult neuronal phenotypes; in addition, there is variability across iPSC lines and differentiation protocols that can affect reproducibility [101,106,107]. Brain organoids partially recapitulate early human cortical development and three-dimensional tissue architecture, but still lack full vascularisation, long-range connectivity, and systemic environmental cues [108,109,110].

### 4.3. Multilineage-Differentiating Stress-Enduring (MUSE) Cells

Multilineage-Differentiating Stress-Enduring (MUSE) cells have emerged as a promising complementary cellular source. MUSE cells were originally described as an endogenous, non-tumorigenic pluripotent stem cell population expressing pluripotency genes such as NANOG, OCT3/4, and SOX2 as well as the surface marker SSEA-3 [111] and found at a low frequency in bone marrow, peripheral blood, adipose tissue, dermis, and other connective tissues [112,113,114]. Estimates suggest that they represent only 0.01–0.03% of bone marrow mononuclear cells, underscoring their scarcity in vivo [111,115]. They were shown to self-renew from single cells and to differentiate spontaneously into derivatives of all three germ layers [111,112]. Unlike conventional pluripotent stem cells, MUSE cells exhibit intrinsic genomic stability, low tumorigenic potential, and the ability to home to damaged tissues [114,116], differentiate into tissue-specific cell types [116,117], and contribute to functional repair [116,117,118], with promising results from heart, brain, lung, and gut injury models [119,120]. The capacity to generate functional neuronal and glial phenotypes from MUSE cells has raised the possibility of positioning them as a stable, physiologically relevant human cell-based platform for investigating neurodevelopmental trajectories and molecular mechanisms underlying psychiatric disorders. MUSE cells isolated from fibroblast cultures can be induced in vitro to generate neural progenitors and subsequently differentiate into dopaminergic, glutamatergic, GABAergic neurons and astrocytes, all implicated in the pathophysiology of major psychiatric disorders such as SCZ, BD, and MDD [121]. Patient-derived MUSE cells carrying pathogenic mutations associated with neurodevelopmental disorders have been shown to exhibit altered neural progenitor formation and biased differentiation toward glial lineages, suggesting that MUSE-based cultures can capture disease-specific developmental abnormalities [122].

However, several technical and practical limitations remain. MUSE cells occur at very low frequency in adult tissues, making their isolation labour-intensive and dependent on specialised purification procedures [111,112]. In addition, expansion and differentiation protocols are expensive and technically demanding, requiring extended culture periods to obtain sufficient numbers of cells for experimental applications [117,123]. Because MUSE-based cellular models are relatively recent compared with iPSC systems, standardised protocols and large-scale comparative validation studies remain limited, particularly in the context of neuropsychiatric disease modelling [123].

## 5. From Adult Neurogenesis to Nasal Access: Unveiling the Olfactory Neuroepithelium as a Human Brain Proxy

In parallel with advances in pluripotent stem-cell technologies, increasing attention has been directed toward endogenous neurogenic niches as alternative sources of human neural cells. Seminal studies demonstrated that neurogenesis persists in discrete regions of the adult mammalian brain, notably the hippocampal dentate gyrus and the subventricular zone of the lateral ventricles, which supplies neuronal precursors to the olfactory bulb [124,125,126]. However, these intracranial niches remain largely inaccessible in living humans, thereby limiting their translational utility. In contrast, the olfactory neuroepithelium (ONE) is a specialised pseudostratified epithelium that, located in the dorsal–posterior region of the nasal cavity, represents a direct extension of the central nervous system beyond the cranial vault [127,128,129]. Pioneering studies on this tissue identified progenitor cells within the olfactory neuroepithelium capable of generating new olfactory sensory neurons throughout adulthood [130]. Subsequent work established that constitutive neurogenesis is sustained by multipotent stem and neural progenitor populations, including globose and horizontal basal cells [129,131,132,133,134]. These cells give rise to olfactory receptor neurons whose axons project through the cribriform plate to the olfactory bulb and, in turn, to limbic and paralimbic regions, such as the hippocampus, amygdala, and entorhinal cortex [135,136,137]. This neuroanatomical continuity provides a compelling biological rationale for using ONE-derived cells as proxies of central nervous system function, [138,139].

The ONE exhibit lifelong regenerative capacity supported by tightly regulated neurogenic programmes involving EGF and FGF2 signalling cascades that recapitulate key aspects of embryonic brain development [140,141,142,143]. Importantly, ONE-derived neural stem and progenitor cells retain lineage commitment as well as donor-specific genetic, epigenetic, and molecular signatures, thereby preserving biologically relevant features of the native tissue [144,145].

A major advantage of the ONE model lies in its accessibility. Historically, ONE tissue was obtained from autopsy specimens or from patients undergoing routine nasal surgery or through biopsies performed under general or local anaesthesia, typically from the nasal septum or superior turbinate [40,141,146,147]. More recently, minimally invasive nasal exfoliation techniques have been developed, enabling safer, rapid, low-cost, and reproducible sampling of ONE cells from living individuals without anaesthesia, demonstrating high tolerability and thereby supporting longitudinal and large-cohort applications [42,44,45,129,148,149,150].

Following collection, ONE-derived neural progenitor cells can be expanded in vitro either as adherent cultures or neurospheres under serum-free conditions supplemented with mitogens such as EGF and FGF2 [128,151]. In the latter case, neurospheres represent a hallmark of neural stem cell behaviour, supporting self-renewal and multilineage differentiation into neurons, astrocytes, and oligodendrocytes, enabling high-throughput genomic, transcriptomic, and proteomic analyses [152,153,154]. Although cellular heterogeneity—due to the presence of respiratory epithelium and non-neural components—remains a limitation, this can be mitigated through approaches such as laser-capture microdissection or magnetic-activated cell sorting [129,155].

ONE-derived models have yielded important insights into the cellular and molecular underpinnings of neuropsychiatric disorders. In SCZ, convergent evidence indicates dysregulated neurodevelopmental processes, including altered cell cycle kinetics, increased proliferation, reduced mitochondrial ATP production, and impaired cell adhesion and migration, potentially linked to aberrant focal adhesion signalling and integrin function [40,44,45,151,156,157]. Transcriptomic studies further implicate key developmental pathways, including Wnt and Notch signalling, which are critical for cell fate determination [45,158,159,160]. Neurons differentiated from ONE-derived neurospheres exhibit altered expression of genes involved in synaptic function and extracellular matrix organisation, such as SCG2, L1CAM, NPTXR, and PTN [161].

Beyond SCZ, ONE-based models have provided insights into BD and MDD. In BD, patient-derived ONE cells display altered adhesion, morphology, and migration, with distinct cellular phenotypes associated with lithium responsiveness and linked to molecular pathways involving CDKN2A, CRMP1, and GSK-3β [131,162,163]. In MDD, ONE-derived cells reveal signatures consistent with impaired neuroplasticity—as evidenced by the reduced expression of BDNF and GSK3 [164] and increased inflammatory markers, including elevated levels of interleukin (IL)-6, IL-8, Thrombospondin-1, and Monocyte Chemoattractant Protein-1 [165]. Collectively, these findings underscore the value of the ONE as a uniquely accessible, biologically informative, and translationally relevant model for investigating the neurodevelopmental and molecular bases of psychiatric disorders.

## 6. Conclusions

In this review, we provide an integrated overview of the principal experimental models used in biological psychiatry, as summarised in Table 1, which synthesises their key characteristics, strengths, and limitations. The evidence presented here points to a paradigm shift in biological psychiatry toward integrated, multi-level approaches and to a progressive evolution of experimental models, in which each methodological advance has laid the groundwork for the next. Rather than emerging as isolated strategies, post-mortem studies, in vivo imaging, animal models, and human-derived cellular systems reflect a continuous and iterative trajectory of discovery. Early post-mortem investigations provided the first direct evidence of molecular and cellular alterations in the human brain, shaping foundational neurobiological hypotheses. These, in turn, enabled the dissection of dynamic processes and causal mechanisms while simultaneously revealing intrinsic limitations in capturing the complexity and specificity of human psychiatric disorders. The subsequent emergence of pluripotent stem-cell technologies marked a critical transition toward patient-specific and developmentally informed models, yet also exposed challenges related to cellular maturity, epigenetic fidelity, and the environmental context.

Within this evolving landscape, the ONE is a uniquely positioned model, offering an unprecedented combination of accessibility, neurobiological relevance, and preservation of patient-specific molecular signatures. While no single model fully captures the complexity of psychiatric disorders, ONE-derived cells provide a critical bridge between systemic and cellular levels of investigation, enabling the study of dynamic, disease-relevant processes directly in living individuals. Future progress will depend on the systematic integration of ONE-based platforms with pluripotent stem-cell technologies, multi-omics approaches, and longitudinal clinical data, thereby fostering a more precise and mechanistically grounded understanding of psychiatric disorders. Such convergence holds promise not only for elucidating core pathogenic mechanisms but also for accelerating the development of biomarkers and targeted, disease-modifying therapies in precision psychiatry.

## 7. Limitations

We acknowledge that this review has several limitations. As a narrative rather than systematic synthesis, it did not apply fully exhaustive, pre-defined selection criteria and may therefore be subject to selection bias. In addition, no formal quality assessment of the included studies was performed, limiting the ability to account for methodological heterogeneity. The focus on the ONE reflects the specific scope of this work and may result in a relative imbalance in the depth of coverage across model systems. Finally, given the rapid evolution of cellular models in psychiatry, some aspects discussed here may require ongoing revision. These considerations underscore the need for future systematic and quantitative syntheses as the field continues to mature.

## Figures and Tables

**Table 1:** Comparative overview of experimental models used in biological psychiatry. PMI = post-mortem interval; hESC = human embryonic stem cell; iPSC = induced pluripotent stem cell; MUSE = multilineage-differentiating stress-enduring; ONE = olfactory neuroepithelium; CNS = central nervous system.

Model	Biological Relevance	Accessibility/Scalability	Patient-Specificity	Key Strengths	Key Limitations
Post-mortem brain	Very high (direct human tissue)	Low (limited samples, autopsy)	Partial (donor)	Direct pathological evidence; established protocols	Static; confounded by PMI, drugs, agonal state; no dynamic processes
Animal models (rodent/primate)	Moderate (rodent) to high (primate)	Moderate (rodents)/Low (primates)	None (non-human)	Longitudinal; causal mechanistic studies; pharmacological screening	Evolutionary gap; poor recapitulation of complex psychiatric symptoms
hESCs	High (human neural lineages)	Moderate–High (scalable)	None (allogeneic)	Scalable; drug screening; developmental studies	Ethical concerns; tumorigenic risk; no patient-specific modelling; allogeneic
iPSCs	High (patient-derived neurons)	Moderate (costly, slow)	High	Patient-specific; models all neural subtypes; genome editing compatible	Epigenetic memory; foetal-stage maturity; variability; costly quality control
MUSE cells	High (endogenous human progenitors)	Low (rare, isolation challenging)	High	Non-tumorigenic; genomic stability; multilineage potential	Very low frequency in tissues; limited standardised protocols in psychiatry
Olfactory neuroepithelium (ONE)	High (CNS proxy; live tissue)	High (minimally invasive, nasal exfoliation)	High	Living donor; longitudinal; large cohorts; genetic/epigenetic preservation	Cellular heterogeneity; limited neuronal maturation in vitro; requires standardisation

## Data Availability

No new data were created or analysed in this study. Data sharing is not applicable to this article.
